# Association of *HOTAIR* rs2366152 and rs1899663 polymorphisms with colorectal cancer susceptibility in Iranian population: A case–control study

**DOI:** 10.1002/jcla.24931

**Published:** 2023-06-20

**Authors:** Nasim Eivazi, Reza Mirfakhraie, Ehsan Nazemalhosseini Mojarad, Javad Behroozi, Vahid Reza Yassaee, Mohammad Tahmaseb, Hossein Sadeghi

**Affiliations:** ^1^ Department of Cellular and Molecular Biology, Faculty of Biological Sciences Kharazmi University Tehran Iran; ^2^ Department of Medical Genetics, Faculty of Medicine Shahid Beheshti University of Medical Sciences Tehran Iran; ^3^ Gastroenterology and Liver Diseases Research Center, Research Institute for Gastroenterology and Liver Diseases Shahid Beheshti University of Medical Sciences Tehran Iran; ^4^ Cancer Epidemiology Research and Treatment Center AJA University of Medical Sciences Tehran Iran; ^5^ Genomic Research Center Shahid Beheshti University of Medical Sciences Tehran Iran

**Keywords:** colorectal cancer, *HOTAIR*, long noncoding RNA, rs1899663, rs2366152

## Abstract

**Background:**

Despite the fact that numerous studies have investigated the association between genetic polymorphisms and colorectal cancer (CRC), more research is required to comprehend the molecular mechanisms of CRC. In the present study, we investigated the association between lncRNA *HOTAIR* rs2366152 and rs1899663 polymorphisms with CRC susceptibility in the Iranian population.

**Methods:**

This case–control study consisting of 187 CRC patients and 200 healthy samples. The tetra‐amplification refractory mutation system‐polymerase chain reaction (Tetra‐ARMS‐PCR) technique was used for the genotyping of rs2366152 and rs1899663 polymorphisms.

**Results:**

The findings showed that the AG genotype of the rs2366152 polymorphism has a protective effect on CRC susceptibility (OR = 0.60, 95% CI: 0.38–0.94, *p*‐value = 0.023). Furthermore, rs2366152 polymorphism associated with CRC risk in an over dominant inheritance model (*p*‐value = 0.0089). According to the outcomes of the rs1899663 polymorphism, the GT genotype had protective effects on CRC risk (OR = 0.55, 95% CI: 0.35–0.86, *p*‐value = 0.008). Moreover, statistical analysis has shown that the rs1899663 polymorphism was associated with CRC risk in dominant (*p*‐value = 0.013) and overdominant (*p*‐value = 0.0086) inheritance models in the Iranian population.

**Conclusion:**

This study confirmed that *HOTAIR* rs2366152 and rs1899663 polymorphisms associated with CRC risk in different inheritance models. It is indeed necessary to do additional research to verify our findings.

## Graphical abstract

After taking samples from patients and healthy people, these samples were subjected to DNA extraction. Then the primer was designed to perform Tetra‐ARMS‐PCR. The obtained results were confirmed by gel electrophoresis and Sanger sequencing. Finally, statistical analysis was done.

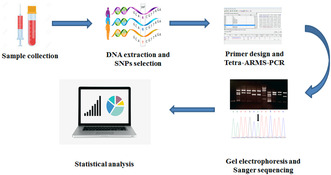

## 1 INTRODUCTION

Colorectal cancer (CRC) is one of the most commonly diagnosed cancers in the world. Epidemiological studies have shown that CRC is third in terms of prevalence, with more than 1,900,000 new cases, and second in terms of mortality, with 935,000 deaths.^1^ In the Iranian population, CRC is the fourth most widespread cancer in males and the third most prevalent cancer in women.^2^ According to the pathophysiology of colon cancer, an accumulation of genetic abnormalities, either somatic or germline, causes the normal colonic epithelium to change into a precancerous lesion and ultimately into an invasive carcinoma. Moreover, genetic alterations can cause cancer cells to undergo apoptotic cell death.^3^, ^4^ Numerous risk factors can alter the prevalence of CRC, according to previous research. In addition, there are established strong connections between environmental or genetic factors and the occurrence of cancer.^5^, ^6^ Furthermore, dietary and lifestyle risk factors, including high amounts of red and processed meat in the diet, low fiber diet, obesity, low physical activity, alcohol consumption, and smoking, may increase CRC incidence.^7^

Researchers have discovered some molecules, such as long noncoding RNA (lncRNA), that play a significant role in the progression of CRC tumorigenesis, despite the fact that the molecular basis of CRC is not entirely known.^8^, ^9^, ^10^ LncRNAs are transcribed by RNA polymerase II and have more than 200 nucleotides.^11^ They have vital functions in regulating chromatin dynamics, gene expression, cellular growth, cell development, and differentiation.^12^ The *HOX Transcript Antisense Intergenic RNA* (*HOTAIR*), which is spliced and polyadenylated, is a well‐known lncRNA. LncRNA *HOTAIR* is transcribed from the antisense strand of the *HOXC* gene cluster and can also alter *HOXD* gene expression.^13^ LncRNA *HOTAIR* in the combination of polycomb repressive complex 2 (PRC2) and LSD1/REST/COREST complex, in 5՛ and 3՛ region, respectively, has involved in chromatin regulation and takes part in histone modifications like H3K27 methylation and H3K4 demethylation.^14^

Studies have demonstrated the role of *HOTAIR* in the pathogenesis of multifactorial diseases. For example, variations in the *HOTAIR* gene increased the risk of recurrent spontaneous abortion (RSA),^15^ and bipolar disorder (BD).^16^ *HOTAIR* may also affect type 2 diabetes mellitus (T2DM) susceptibility by modulating various signaling pathways.^17^ In addition, studies have shown the *HOTAIR* gene contributes to tumorigenesis^18^ and is associated with various cancers' susceptibility, including CRC.^19^, ^20^, ^21^ Despite cancer's development, various therapeutic methods are being discovered for cancer treatment.^22^, ^23^ However, the molecular pathway of cancer is still not completely understood. Hence, more studies on the cancer disease are required.

A well‐known genetic variant is single nucleotide polymorphism (SNP), which has different roles in cancer susceptibility based on its region. For instance, SNPs in the exons change gene transcription and translation, while SNPs in the introns have roles in RNA splicing, genomic imprinting, and also the function of lncRNAs.^24^ The functional SNP rs4759314 A>G in lncRNA *HOTAIR* is associated with an increased risk of gastric cancer in the Chinese population. LncRNA *HOTAIR* polymorphism rs4759314 is located in the intronic promoter region, changes promoter activity, and contributes to potential mechanisms for gastric cancer susceptibility by affecting the expression of *HOTAIR* and *HOXC11* genes.^25^ Wang et al.^26^ discovered that the rs1899663 G>T polymorphism on the lncRNA *HOTAIR* increases the risk of lung cancer. The Chinese population has been studied for the SNP rs1899663 G>T in the lncRNA *HOTAIR*. According to the findings, rs1899663 G>T was associated with breast cancer, and the T allele frequency in cases was higher than in controls. More analysis also showed that TT carriers of rs1899663 were associated with an increased risk of breast cancer.^27^ Sharma Saha et al.^28^ have shown the C allele of rs2366152 is prevalent among cervical cancer cases that lead to low *HOTAIR* expression. Taken together, according to the meta‐analysis study, multiple SNPs are already associated with various kinds of cancer.^29^

The association between the lncRNA *HOTAIR* rs1899663 and rs2366152 and CRC has not been studied in the Iranian population yet. As a result, our case–control study sought to assess the association between the lncRNA *HOTAIR* rs2366152 and rs1899663 polymorphisms and CRC susceptibility in Iranian population.

## 2 MATERIALS AND METHODS

### 2.1 Subjects

In our case–control study, we evaluated 387 participants, comprising 200 healthy controls and 187 colorectal cancer cases which were referred from Taleghani Hospital. First, clinical interviews and medical records, were used to collect some information, such as the age, gender, stage, grade of tumors, family history of cancer, and marital status of participants. Additionally, based on their patients' histories, which included information from clinical evaluations and colonoscopies, the confirmation of every affected person who had been diagnosed with CRC was checked. All participants signed informed consent forms after being informed of the study's goals. The study protocol was confirmed by the ethics committee of the Shahid Beheshti University of Medical Sciences (IR.SBMU.RETECH.REC.1401.384).

### 2.2 DNA extraction and genotyping

Peripheral blood samples were collected from all subjects, and genomic DNA was then extracted using the Puregene® Blood Core Kit C (Qiagen). We used the tetra‐amplification refractory mutation system‐polymerase chain reaction (Tetra‐ARMS‐PCR) method for genotyping.^30^ We used Primer1 online software to design specific primers for each SNP.^31^ The Applied Biosystems thermocycler (ABI) was used to perform the Tetra‐ARMS‐PCR reaction. Each sample had a final volume of 25 μL, contained 2 μL of genomic DNA (100–200 ng), 12.5 μL Taq DNA polymerase 2× master mix Red (Amplicon), 1 μL of each primer (10 pmol), and 6.5 μL of PCR grade water. PCR conditions comprise initial denaturation for 5 min at 95°C, followed by denaturation at 95°C for 1 min and annealing for 50 s at the temperature exhibited in Table 1, and an extension step for 1 min and 20 s at 72°C, throughout the 35‐cycle period. The final extension was done for 5 min at 72°C. The primer sequences and annealing temperatures for the rs2366152 and rs1899663 polymorphisms are included in Table 1. Following amplification, PCR products were separated using 2% agarose gel electrophoresis with DNA gel stain in 0.5× tris/borate/EDTA (TBE).

**TABLE 1:** Primer sequences and their characteristics for amplification of rs2366152 and rs1899663 polymorphisms.

SNP	Primer	Primer sequence	Amplicon size (bp)	*T*_a_
Rs2366152	FI	TGTGTGTGTACTAAATACATTGAAATTTCG	196 (G allele)	56
	RI	TTAAACAGTTCATGAACCCAGAAGCAT	262 (A allele)	
	FO	AATGAATGTGCAGTCCTGAGTCTATTT	401	
	RO	GTATGTAGACACAGAAGGGGTATTTAAA		
Rs1899663	FI	CCATTTTTCCAGTTGAGGAGGGTGAAT	226 (T allele)	59
	RI	TCCAAAAGCCTCTAATTGTTGTCGCC	281 (G allele)	
	FO	AAGCCAGGATCATTTAACATCACCAGAAA	454	
	RO	TATCTGCTGAGGACTTACCTTTTTCCTG		

Abbreviations: bp, base pair; F, forward; I, inner; O, outer; R, reverse; *T*_a_; annealing temperature.

### 2.3 Bioinformatical analysis

To determine the possible biological role of *HOTAIR* polymorphisms and to investigate the alteration of the binding affinity of transcription factors at *HOTAIR* polymorphism locus, we carried out in silico analysis using HaploReg,^32^ RegulomeDB,^33^ ORegAnno,^34^ and PROMO^35^ online tools.

### 2.4 Statistical analysis

In order to calculate allele and genotype frequencies based on the Hardy–Weinberg equilibrium, we used *χ* ^2^ test in SNPStats^36^ and MEDCALC^37^ online software. Statistical analysis evaluated the association of *HOTAIR* rs2366152 and rs1899663 polymorphisms with CRC in recessive, dominant, overdominant, and log‐additive inheritance models. A statistically significant *p*‐value was regarded as being <0.05.

## 3 RESULTS

**FIGURE 1:**
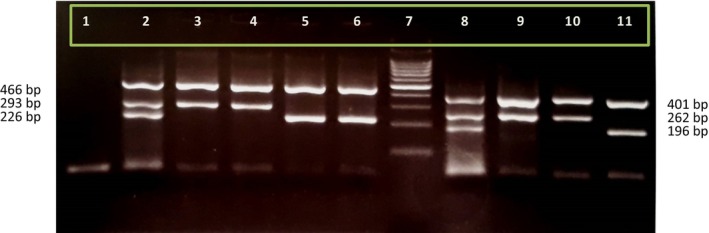
The representation of 2% agarose gel electrophoresis for determining HOTAIR rs1899663 and rs2366152 genotypes. Lane 1: Negative control, lane 2: GT genotype of rs1899663 polymorphism, lanes 3 and 4: GG genotype of rs1899663, lanes 5 and 6: TT genotype of rs1899663, lane 7: 100 bp + 3k DNA ladder, lane 8: AG genotype of rs2366152 polymorphism. Lanes 9 and 10: AA genotype of rs2366152 and lane 11: GG genotype of rs2366152 polymorphism.

**FIGURE 2:**
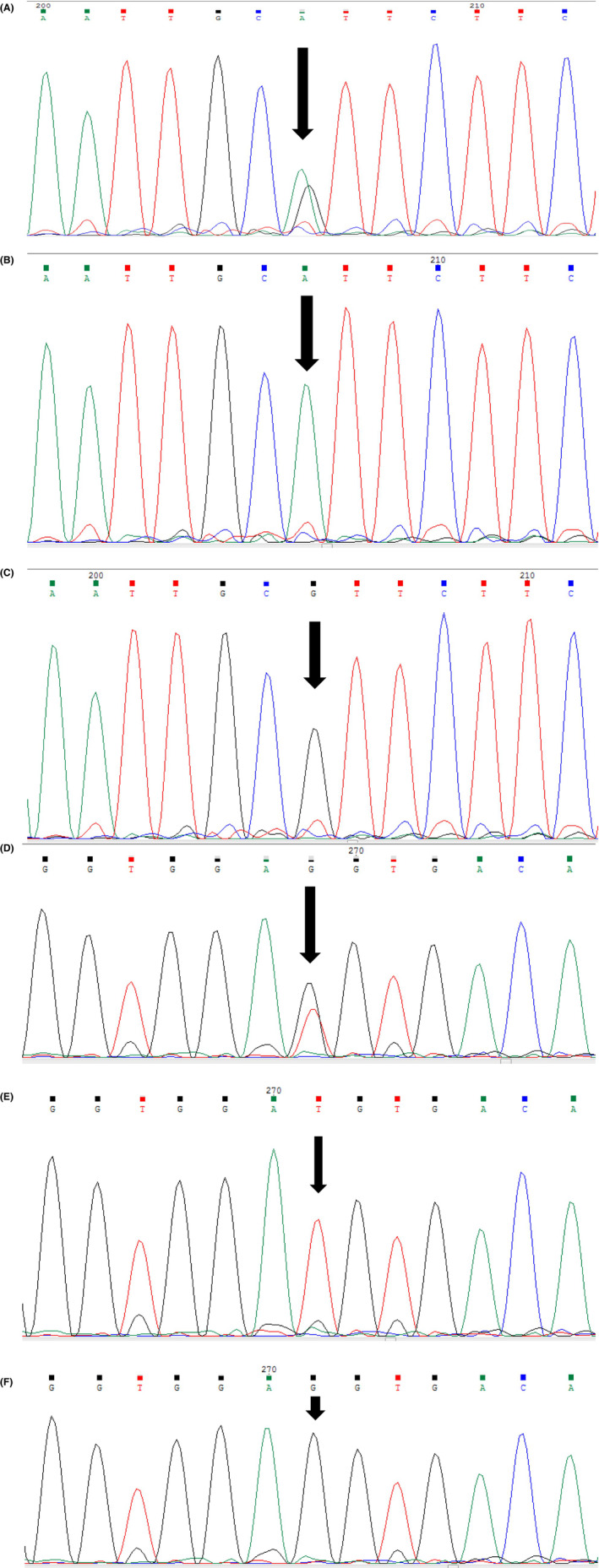
The electropherograms represent the rs2366152 and rs1899663 genotypes. (A–C) Show the AG, AA, and GG genotypes of the rs2366152 polymorphism, respectively. Also, the electrophoregrams for the rs1899663 polymorphism's GT, TT, and GG genotypes are shown in (D–F), respectfully.

Genotypes and allele frequencies of the rs2366152 and rs1899663 SNPs in the cases and healthy groups are shown in Table 2. Case and control groups in both SNPs were not in Hardy–Weinberg equilibrium. The association between *HOTAIR* rs2366152 and rs1899663 polymorphisms and CRC susceptibility was studied in recessive, dominant, overdominant, and log‐additive inheritance models. Statistical analysis demonstrated that in the polymorphic variant rs2366152, the A/G genotype had a protective effect on CRC susceptibility (OR = 0.60, 95% CI: 0.38–0.94, *p* = 0.023). Moreover, in the overdominant inheritance model, A/A + G/G versus A/G genotypes were statistically associated with CRC risk and also decreased CRC susceptibility (OR = 0.57, 95% CI: 0.37–0.87, *p* = 0.0089). In dominant inheritance model, A/G − G/G versus A/A genotypes were not associated with CRC susceptibility (OR = 0.65, 95% CI: 0.42–1.01, *p* = 0.056). Polymorphism rs1899663 G>T is also studied in 187 CRC cases and 200 non‐cancer samples. Although the observed differences in rs1899663T allele frequency were not statistically significant between case and control samples, statistical analysis showed that G/T genotype was associated with CRC susceptibility and had a protective effect on CRC risk (OR = 0.55, 95% CI: 0.35–0.86, *p*‐value = 0.008). Furthermore, in the dominant inheritance model, G/T − T/T versus G/G genotypes are associated with CRC risk (OR = 0.57, 95% CI: 0.37–0.89, *p*‐value = 0.013). Also, statistical analysis showed that in the overdominant inheritance model, G/G − T/T versus G/T genotypes are associated with CRC risk (OR = 0.57, 95% CI = 0.38–0.87, *p*‐value = 0.0086). Taken together, statistical analysis explained how the rs1899663 polymorphism decreased CRC risk in the dominant and overdominant inheritance models. *HOTAIR* rs2366152 and rs1899663 genotype electrophoresis on 2% agarose gel is shown in Figure 1. Also, Figure 2 represents the electropherogram of both *HOTAIR* polymorphisms. Table 3 describes the demographic variables and clinical characteristics of the studied subjects.

**TABLE 2:** Genotypes and allele frequencies of polymorphisms rs2366152 and rs1899663 in colorectal cancer patients and non‐cancer controls.

Inheritance models	Genotype/allele	Case, *N* (%)	Control, *N* (%)	OR (95% CI)	*p*‐Value
Rs2366152 A>G					
Codominant	A/A	71 (40.3)	55 (30.6)	1.00	
	A/G	90 (51.1)	117 (65)	0.60 (0.38–0.94)	0.023
	G/G	15 (8.5)	8 (4.4)	1.44 (0.57–3.64)	0.428
Dominant	A/A vs. A/G + G/G	71 (40.3)	55 (30.6)	1.00	
		105 (59.7)	125 (69.4)	0.65 (0.42–1.01)	0.056
Recessive	A/A + A/G vs. G/G	161 (91.5)	172 (95.6)	1.00	
		15 (8.5)	8 (4.4)	1.97 (0.81–4.78)	0.12
Overdominant	A/A + G/G vs. A/G	86 (48.9)	63 (35)	1.00	
		90 (51.1)	117 (65)	0.57 (0.37–0.87)	0.0089
Log‐additive	…	…	…	0.84 (0.59–1.21)	0.35
	A	232 (0.66)	227 (0.63)	0.88 (0.64–1.2)	0.42
	G	120 (0.34)	133 (0.37)	0.88 (0.64–1.2)	0.42
Rs1899663 G>T					
Codominant	G/G	69 (38.1)	50 (26.2)	1.00	
	G/T	95 (52.5)	126 (66)	0.55 (0.35–0.86)	0.008
	T/T	17 (9.4)	15 (7.8)	0.81 (0.37–1.77)	0.622
Dominant	G/G vs. G/T + T/T	69 (38.1)	50 (26.2)	1.00	
		112 (61.9)	141 (73.8)	0.57 (0.37–0.89)	0.013
Recessive	G/G + G/T vs. T/T	164 (90.6)	176 (92.2)	1.00	
		17 (9.4)	15 (7.8)	1.20 (0.58–2.48)	0.63
Overdominant	G/G + T/T vs. G/T	86 (47.5)	65 (34)	1.00	
		95 (52.5)	126 (66)	0.57 (0.38–0.87)	0.0086
Log‐additive	…	…	…	0.74 (0.52–1.04)	0.085
	G	233 (0.64)	226 (0.59)	0.802 (0.59–1.07)	0.14
	T	129 (0.36)	156 (0.41)	0.802 (0.59–1.07)	0.14

Abbreviations: CI, confidence interval; OR, odds ratio.

**TABLE 3:** Demographic data and clinical characteristics of the study participants.

Characteristics	CRC (*n* = 187) patients	Non‐cancer controls (*n* = 200)
Median age, years	56.89 ± 9.24	55.18 ± 10.36
Primary tumor location		
Colon	61%	
Rectum	27%	
Cecum	12%	
Differentiation		
Well differentiated	39%	
Moderately differentiated	20%	
Poorly differentiated	10%	
Not determined	31%	
Cigarette smoking		
No	71%	
Yes	29%	
Clinical stages, TNM		
I	15%	
II	42%	
III	25%	
IV	18%	

## 4 DISCUSSION

According to recent studies, although extensive investigations have been completed about the association between CRC and polymorphisms on lncRNAs having a vital function in growth, metastasis, and treatment, the precise molecular mechanism of CRC is not entirely understood.^38^, ^39^, ^40^ Numerous studies have concentrated on the association between SNPs in the lncRNA *HOTAIR* and cancer susceptibility.^40^, ^41^, ^42^, ^43^, ^44^, ^45^ However, the association between the *HOTAIR* polymorphisms rs2366152 and rs1899663 and the risk of CRC is not fully understood.

Our results confirmed that the heterozygote genotype (A/G) of rs2366152 SNP associated with CRC susceptibility (*p* = 0.023) and had protective effects on CRC risk. Furthermore, rs2366152 A>G significantly associated with susceptibility to CRC in an overdominant inheritance model (*p* = 0.0089). In this research, in silico analysis has been done by using the HaploReg,^32^ RegulomeDB,^33^ and PROMO^35^ online tools. Chip‐seq data illustrated that EZH2, SUZ12, and CBX8 as components of PRC2 have binding affinity for the rs2366152 locus. High EZH2 expression was shown to be substantially correlated with tumor stage, tumor size, histological differentiation, and lymph node metastasis of colon cancer, according to an investigation of the relationship between clinicopathological characteristics and EZH2 expression.^46^ Additionally, HaploReg^32^ demonstrated that the rs2366152 SNP may alter the TEF‐1 and CEBPD transcription factors' binding sites, and according to PROMO^35^ online tool, the rs2366152 polymorphism could alter the POU2F1 transcription factor's binding affinity. Related to our results, Saha et al. showed that the C allele of the rs2366152 polymorphism is associated with low *HOTAIR* expression in cervical cancer. They also discovered that the C allele of rs2366152 could affect *HOTAIR* expression levels, particularly in cases of low *HOTAIR* expression cervical cancer.^28^

In the present study, we have also analyzed the association between rs1899663 and CRC risk. Although statistical analysis demonstrated that there were no significant differences in allele frequencies between CRC cases and control groups, it showed that the G/T genotype in the rs1899663 polymorphism is associated with CRC risk (*p*‐value = 0.008) and has protective effects on CRC susceptibility. Furthermore, the rs1899663 polymorphism is associated with CRC susceptibility and decreased CRC risk in dominant (*p*‐value = 0.013) and overdominant (*p*‐value = 0.0086) inheritance models. Consistent with our study, Kim et al. demonstrated that the rs1899663 heterozygote genotype was significantly different between case and control groups, and also the rs1899663 polymorphism was associated with CRC susceptibility in the dominant model of inheritance.^21^

Another study in the Iranian population found that the rs1899663 T allele was associated with the risk of benign prostate hyperplasia, as well as cancer risk in recessive, dominant, and codominant inheritance models.^47^ Investigations of the association between the rs1899663 SNP and lung cancer risk in the Chinese population showed that the heterozygote genotype of the rs1899663 site increased the susceptibility to lung cancer compared with the G/G genotype.^26^ According to an association study between the rs1899663 polymorphism and breast cancer in the Iranian population, the rs1899663 G>T variant significantly decreased the risk of breast cancer in dominant, codominant, and overdominant inheritance models.^48^ On the other hand, Tian et al. explained in a meta‐analysis that there was no significant association between the rs1899663 polymorphism and cancer risk.^49^

In silico analysis, PROMO^35^ online tool, indicated that the rs1899663 polymorphism may alter the binding affinities of numerous transcription factors, like PEA3, T3R‐beta1, GR‐alpha, and LyF‐1. Furthermore, according to the Haploreg^32^ online tool, the binding sites of the transcription factors PAX4‐3, Sox15, SPZ1‐1, and Zfp281 may be affected by the rs1899663 polymorphism. Wang et al. demonstrated that the expression of the SPZ‐1 transcription factor could exhibit a tumor‐specific expression pattern and a high correlation with tumor size, tumor number, and progression stage, which were studied in hepatocarcinoma cells.^50^

In conclusion, we studied the association between *HOTAIR* rs2366152 and rs1899663 polymorphisms with CRC risk in the Iranian population for the first time. There are certain limitations to the current research, namely the small number of samples. As a result, confirming with a greater number of samples is necessary. Second, the association between *HOTAIR* polymorphisms and CRC susceptibility was investigated in the current study. Extensive research is needed to fully understand how SNPs affect clinical decisions regarding colorectal cancer. Furthermore, our findings need to be confirmed by other research in other populations.

## FUNDING INFORMATION

Genomic Research Center, Shahid Beheshti University of Medical Sciences.

## CONFLICT OF INTEREST STATEMENT

The authors declare that there is no conflict of interest.

## INFORMED CONSENT

The informed consent was taken from all the patients who participated in this study.

## Data Availability

The data that support the findings of this study are available from the corresponding author upon reasonable request.

## References

[1] Morgan E , Arnold M , Gini A , et al. Global burden of colorectal cancer in 2020 and 2040: incidence and mortality estimates from GLOBOCAN. Gut. 2023;72(2):338‐344. 36604116 10.1136/gutjnl-2022-327736

[2] Rafiemanesh H , Pakzad R , Abedi M , et al. Colorectal cancer in Iran: epidemiology and morphology trends. EXCLI J. 2016;15:738‐744. 28337105 10.17179/excli2016-346 PMC5318687

[3] Bien SA , Su YR , Conti DV , et al. Genetic variant predictors of gene expression provide new insight into risk of colorectal cancer. Hum Genet. 2019;138(4):307‐326. 30820706 10.1007/s00439-019-01989-8 PMC6483948

[4] Mollashahee‐Kohkan F , Saravani R , Khalili T , Galavi H , Sargazi S . Levisticum officinale extract triggers apoptosis and down‐regulates ZNF703 gene expression in breast cancer cell lines. Rep Biochem Mol Biol. 2019;8(2):119‐125. 31832434 PMC6844619

[5] Parsa N . Environmental factors inducing human cancers. Iran J Public Health. 2012;41(11):1‐9. PMC3521879 23304670

[6] Harati‐Sadegh M , Sargazi S , Saravani M , Sheervalilou R , Mirinejad S , Saravani R . Relationship between miR‐143/145 cluster variations and cancer risk: proof from a meta‐analysis. Nucleosides Nucleotides Nucleic Acids. 2021;40(5):578‐591. 33980135 10.1080/15257770.2021.1916030

[7] Sameer A . Colorectal cancer: molecular mutations and polymorphisms. Front Oncol. 2013;3:3. 23717813 10.3389/fonc.2013.00114 PMC3651991

[8] Wang J , Song YX , Ma B , et al. Regulatory roles of non‐coding RNAs in colorectal cancer. Int J Mol Sci. 2015;16(8):19886‐19919. 26307974 10.3390/ijms160819886 PMC4581331

[9] Liu Y , Chen X , Chen X , et al. Long non‐coding RNA HOTAIR knockdown enhances radiosensitivity through regulating microRNA‐93/ATG12 axis in colorectal cancer. Cell Death Dis. 2020;11(3):175. 32144238 10.1038/s41419-020-2268-8 PMC7060216

[10] Huang Y , Wang L , Liu D . HOTAIR regulates colorectal cancer stem cell properties and promotes tumorigenicity by sponging miR‐211‐5p and modulating FLT‐1. Cell Cycle. 2021;20(19):1999‐2009. 34470574 10.1080/15384101.2021.1962636 PMC8565839

[11] Kurokawa R . Long noncoding RNA as a regulator for transcription. In: Ugarkovic D , ed. Long Non‐Coding RNAs. Springer; 2011:29‐41. 10.1007/978-3-642-16502-3_2 21287132

[12] Bhan A , Mandal SS . LncRNA HOTAIR: a master regulator of chromatin dynamics and cancer. Biochim Biophys Acta. 2015;1856(1):151‐164. 26208723 10.1016/j.bbcan.2015.07.001 PMC4544839

[13] Rinn JL , Kertesz M , Wang JK , et al. Functional demarcation of active and silent chromatin domains in human HOX loci by noncoding RNAs. Cell. 2007;129(7):1311‐1323. 17604720 10.1016/j.cell.2007.05.022 PMC2084369

[14] Tsai MC , Manor O , Wan Y , et al. Long noncoding RNA as modular scaffold of histone modification complexes. Science. 2010;329(5992):689‐693. 20616235 10.1126/science.1192002 PMC2967777

[15] Salimi S , Sargazi S , Heidari Nia M , Mirani Sargazi F , Ghasemi M . Genetic variants of HOTAIR are associated with susceptibility to recurrent spontaneous abortion: a preliminary case–control study. J Obstet Gynaecol Res. 2021;47(11):3767‐3778. 34396639 10.1111/jog.14977

[16] Sargazi S , Zahedi Abghari A , Mirinejad S , et al. Long noncoding RNA HOTAIR polymorphisms and susceptibility to bipolar disorder: a preliminary case–control study. Nucleosides Nucleotides Nucleic Acids. 2022;41(7):684‐701. 35469536 10.1080/15257770.2022.2065017

[17] Sargazi S , Ravanbakhsh M , Nia MH , et al. Association of polymorphisms within HOX transcript antisense RNA (HOTAIR) with type 2 diabetes mellitus and laboratory characteristics: a preliminary case–control study. Dis Markers. 2022;2022:4327342. 35359879 10.1155/2022/4327342 PMC8964191

[18] Kogo R , Shimamura T , Mimori K , et al. Long noncoding RNA HOTAIR regulates polycomb‐dependent chromatin modification and is associated with poor prognosis in colorectal cancers. Cancer Res. 2011;71(20):6320‐6326. 21862635 10.1158/0008-5472.CAN-11-1021

[19] Li H , Yang Z , Li J , et al. Genetic variants in lncRNA HOTAIR are associated with lung cancer susceptibility in a Chinese Han population in China: a case–control study. Cancer Manag Res. 2018;10:5209‐5218. 30464618 10.2147/CMAR.S175961 PMC6217179

[20] Kim HJ , Lee DW , Yim GW , et al. Long non‐coding RNA HOTAIR is associated with human cervical cancer progression. Int J Oncol. 2015;46(2):521‐530. 25405331 10.3892/ijo.2014.2758 PMC4277242

[21] Kim JO , Jun HH , Kim EJ , et al. Genetic variants of HOTAIR associated with colorectal cancer susceptibility and mortality. Front Oncol. 2020;10:72. 32117729 10.3389/fonc.2020.00072 PMC7020018

[22] Chakrabarti S , Peterson CY , Sriram D , Mahipal A . Early stage colon cancer: current treatment standards, evolving paradigms, and future directions. World J Gastrointest Oncol. 2020;12(8):808‐832. 32879661 10.4251/wjgo.v12.i8.808 PMC7443846

[23] Barani M , Rahdar A , Mukhtar M , et al. Recent application of cobalt ferrite nanoparticles as a theranostic agent. Mater Today Chem. 2022;26:101131.

[24] Deng N , Zhou H , Fan H , Yuan Y . Single nucleotide polymorphisms and cancer susceptibility. Oncotarget. 2017;8(66):110635‐110649. 29299175 10.18632/oncotarget.22372 PMC5746410

[25] Du M , Wang W , Jin H , et al. The association analysis of lncRNA HOTAIR genetic variants and gastric cancer risk in a Chinese population. Oncotarget. 2015;6(31):31255‐31262. 26384301 10.18632/oncotarget.5158 PMC4741602

[26] Wang C , Li Y , Li YW , et al. HOTAIR lncRNA SNPs rs920778 and rs1899663 are associated with smoking, male gender, and squamous cell carcinoma in a Chinese lung cancer population. Acta Pharmacol Sin. 2018;39(11):1797‐1803. 30154526 10.1038/s41401-018-0083-x PMC6289398

[27] Lin Y , Guo W , Li N , Fu F , Lin S , Wang C . Polymorphisms of long non‐coding RNA HOTAIR with breast cancer susceptibility and clinical outcomes for a southeast Chinese Han population. Oncotarget. 2018;9(3):3677‐3689. 29423075 10.18632/oncotarget.23343 PMC5790492

[28] Sharma Saha S , Roy Chowdhury R , Mondal NR , et al. Identification of genetic variation in the lncRNA HOTAIR associated with HPV16‐related cervical cancer pathogenesis. Cell Oncol (Dordr). 2016;39(6):559‐572. 27683269 10.1007/s13402-016-0298-0 PMC13001853

[29] Sargazi S , Abghari AZ , Sarani H , et al. Relationship between CASP9 and CASP10 gene polymorphisms and cancer susceptibility: evidence from an updated meta‐analysis. Appl Biochem Biotechnol. 2021;193(12):4172‐4196. 34463927 10.1007/s12010-021-03613-w

[30] Medrano RF , de Oliveira CA . Guidelines for the tetra‐primer ARMS‐PCR technique development. Mol Biotechnol. 2014;56(7):599‐608. 24519268 10.1007/s12033-014-9734-4

[31] Collins A . Primer1: primer design web service for tetra‐primer ARMS‐PCR. Open Bioinform. 2012;6:55‐58.

[32] Ward LD , Kellis M . HaploReg v4: systematic mining of putative causal variants, cell types, regulators and target genes for human complex traits and disease. Nucleic Acids Res. 2016;44(D1):D877‐D881. 26657631 10.1093/nar/gkv1340 PMC4702929

[33] Boyle AP , Hong EL , Hariharan M , et al. Annotation of functional variation in personal genomes using RegulomeDB. Genome Res. 2012;22(9):1790‐1797. 22955989 10.1101/gr.137323.112 PMC3431494

[34] Griffith OL , Montgomery SB , Bernier B , et al. ORegAnno: an open‐access community‐driven resource for regulatory annotation. Nucleic Acids Res. 2008;36:D107‐D113. 18006570 10.1093/nar/gkm967 PMC2239002

[35] Messeguer X , Escudero R , Farré D , Núñez O , Martínez J , Albà MM . PROMO: detection of known transcription regulatory elements using species‐tailored searches. Bioinformatics. 2002;18(2):333‐334. 11847087 10.1093/bioinformatics/18.2.333

[36] Solé X , Guinó E , Valls J , Iniesta R , Moreno V . SNPStats: a web tool for the analysis of association studies. Bioinformatics. 2006;22(15):1928‐1929. 16720584 10.1093/bioinformatics/btl268

[37] Schoonjans F , Zalata A , Depuydt CE , Comhaire FH . MedCalc: a new computer program for medical statistics. Comput Methods Programs Biomed. 1995;48(3):257‐262. 8925653 10.1016/0169-2607(95)01703-8

[38] Prensner JR , Chinnaiyan AM . The emergence of lncRNAs in cancer biology. Cancer Discov. 2011;1(5):391‐407. 22096659 10.1158/2159-8290.CD-11-0209 PMC3215093

[39] Gutschner T , Diederichs S . The hallmarks of cancer: a long non‐coding RNA point of view. RNA Biol. 2012;9(6):703‐719. 22664915 10.4161/rna.20481 PMC3495743

[40] Svoboda M , Slyskova J , Schneiderova M , et al. HOTAIR long non‐coding RNA is a negative prognostic factor not only in primary tumors, but also in the blood of colorectal cancer patients. Carcinogenesis. 2014;35(7):1510‐1515. 24583926 10.1093/carcin/bgu055

[41] Martínez‐Fernández M , Feber A , Dueñas M , et al. Analysis of the polycomb‐related lncRNAs HOTAIR and ANRIL in bladder cancer. Clin Epigenetics. 2015;7(1):109. 26457124 10.1186/s13148-015-0141-x PMC4599691

[42] Xu Z‐Y , Yu QM , du YA , et al. Knockdown of long non‐coding RNA HOTAIR suppresses tumor invasion and reverses epithelial–mesenchymal transition in gastric cancer. Int J Biol Sci. 2013;9(6):587‐597. 23847441 10.7150/ijbs.6339 PMC3708039

[43] Qin W , Kang P , Xu Y , et al. Long non‐coding RNA HOTAIR promotes tumorigenesis and forecasts a poor prognosis in cholangiocarcinoma. Sci Rep. 2018;8(1):12176. 30111807 10.1038/s41598-018-29737-4 PMC6093929

[44] Zhang J , Liu X , You LH , Zhou RZ . Significant association between long non‐coding RNA HOTAIR polymorphisms and cancer susceptibility: a meta‐analysis. Onco Targets Ther. 2016;9:3335‐3343. 27330313 10.2147/OTT.S107190 PMC4898434

[45] Alzeer HS , Shaik JP , Reddy Parine N , et al. Genetic variants of HOTAIR associated with colorectal cancer: a case–control study in the Saudi population. Genes. 2023;14(3):592. 36980864 10.3390/genes14030592 PMC10047939

[46] Chen Z , Yang P , Li W , et al. Expression of EZH2 is associated with poor outcome in colorectal cancer. Oncol Lett. 2018;15(3):2953‐2961. 29435024 10.3892/ol.2017.7647 PMC5778885

[47] Taheri M , Habibi M , Noroozi R , et al. HOTAIR genetic variants are associated with prostate cancer and benign prostate hyperplasia in an Iranian population. Gene. 2017;613:20‐24. 28259691 10.1016/j.gene.2017.02.031

[48] Hassanzarei S , Hashemi M , Sattarifard H , Hashemi SM , Bahari G , Ghavami S . Genetic polymorphisms of HOTAIR gene are associated with the risk of breast cancer in a sample of southeast Iranian population. Tumour Biol. 2017;39(10):1010428317727539. 29022495 10.1177/1010428317727539

[49] Tian T , Li C , Xiao J , et al. Quantitative assessment of the polymorphisms in the HOTAIR lncRNA and cancer risk: a meta‐analysis of 8 case–control studies. PLoS One. 2016;11(3):e0152296. 27010768 10.1371/journal.pone.0152296 PMC4806879

[50] Wang LT , Chiou SS , Chai CY , et al. Transcription factor SPZ1 promotes TWIST‐mediated epithelial–mesenchymal transition and oncogenesis in human liver cancer. Oncogene. 2017;36(31):4405‐4414. 28368406 10.1038/onc.2017.69 PMC5543259

